# Antineoplastic and Cytotoxic Activities of Nickel(II) Complexes of Thiosemicarbazones

**DOI:** 10.1155/MBD.1997.89

**Published:** 1997

**Authors:** Iris H. Hall, Merrill C. Miller, Douglas X. West

**Affiliations:** 1 Division of Medicinal Chemistry and Natural Products School of Pharmacy University of North Carolina Chapel Hill North Carolina 27599-7360 USA; 2 Department of Chemistry Illinois State University Normal Illinois 61761 USA

## Abstract

Nickel(II) complexes of thiosemicarbazons were observed to be potent cytotoxic agents in human and rodent tissue cultured tumor cells. Each compound demonstrated a slightly different profile in the various histological types of tumors. The nickel complex of Appip demonstrated the most potent *in vivo* activity in the Ehrlich ascites carcinoma. This agent selectively inhibited L1210 DNA and purine syntheses, and DNA polymerase α, PRPP-amido transferase, IMP-dehydrogenase, dihydrofolate reductase, TMP-kinase and thymidylate synthetase activities. L1210 DNA strand scission was evident and DNA viscosity was reduced after 24 hr incubation. The nickel complexes were not L1210 DNA topoisomerase II inhibitors.


### ANTINEOPLASTIC AND CYTOTOXIC ACTIVITIES OF NICKEL(II) COMPLEXES OF THIOSEMICARBAZONES

Iris H. Hall*[1], Merrill C. Miller, III,[1] and Douglas X. West[2]

[1] Division of Medicinal Chemistry and Natural Products, School of Pharmacy, University of North Carolina, Chapel Hill, North Carolina 27599-7360, USA
[2] Department of Chemistry, Illinois State University, Normal, Illinois, 61761, USA

### Abstract

Nickel(II) complexes of thiosemicarbazons were observed to be potent cytotoxic agents in human and rodent tissue cultured tumor cells. Each compound demonstrated a slightly different profile in the various histological types of tumors. The nickel complex of Appip demonstrated the most potent *in vivo* activity in the Ehrlich ascites carcinoma. This agent selectively inhibited L1210 DNA and purine syntheses, and DNA polymerase α, PRPP-amido transferase, IMP-dehydrogenase, dihydrofolate reductase, TMP-kinase and thymidylate synthetase activities. L1210 DNA strand scission was evident and DNA viscosity was reduced after 24 hr incubation. The nickel complexes were not L1210 DNA topoisomerase II inhibitors.

### Introduction

Copper, nickel and cobalt metal complexes of heterocyclic thiosemicarbazones, thioureas and 2-substituted pyridines have previously been shown to be potent anti-neoplastic agents in the Ehrlich ascites carcinoma screen [1,2]. Cytotoxicity was demonstrated against L1210 and $Tmolt_3$ leukemias, as well as human solid tumor growth, e.g. HeLa, KB nasopharynx, skin, bronchogenic lung carcinomas, bone osteosarcoma and glioma. A mode of action study with copper(II) complexes in L1210 leukemia cells showed that DNA synthesis followed by RNA synthesis was inhibited. The major enzymatic sites of inhibition by the agents include: IMP dehydrogenase, dihydrofolate reductase, DNA polymerase α, ribonucleoside reductase, and nucleoside kinase [1,2]. More important was the observation that DNA strand scission occurred after 24 hr. incubation with the agents. There did not appear to be any evidence of cross linking of the DNA nor did the agents produce intercalation between nucleic bases of DNA. The copper complexes of the heterocyclic thiosemicarbazones, thioureas and 2-substituted pyridines were shown to be L1210 DNA topoisomerase II inhibitors much like the agent fostriecin in that they were competitive blockers of VP-16's induction of DNA protein linked breaks [3-5]. The present investigation involves an in-depth study of the nickel complexes regarding their mode of action in L1210 leukemia cells.

### Methods

All test compounds were previously synthesized and their chemical and physical characteristics reported[6,7], i.e. Compound #1 $[Ni(Appip)]_2$, compound #2 [Ni(pip)Br] and compound #3 $[H_2Appip]$ [Fig 1]. All radioisotopes were purchased from New England Chemical Co. (St. Louis, MO). Radioactivity was determined in Fisher Scintiverse scintillation fluid with correction for quenching. Substrates and cofactors were obtained from Sigma Chemical Co.

**Pharmacological methods**

Compounds **1** and **2** (Table 1) were tested for cytotoxic activity by homogenizing drugs in a 1 mM solution in 0.05% Tween 80/$H_2O$. These solutions were sterilized by passing them through an acrodisc (0.45 μ). The following cell lines were maintained by literature techniques[1,2]: murine L1210 lymphoid leukemia, human $Tmolt_3$ acute lymphoblastic T cell leukemia, colorectal adenocarcinoma SW480, lung bronchogenic MB-9812, osteosarcoma TE418, KB epidermoid nasopharynx, HeLa-$S^3$ suspended cervical carcinoma, and glioma EH 118 MG. Geran et al.'s protocol [8] was used to assess the cytotoxicity of the compounds and standards in each cell line. Values for cytotoxicity were expressed as ED50 = μg/ml, i.e.

the concentration of the compound inhibiting 50% of cell growth. ED50 values were determined by the trypan blue exclusion technique. A value of less than 4 μg/ml was required for significant activity of growth inhibition. Standard agents, e.g. 5-fluorouracil [5-FU], 6-mercaptopurine [6-MP], cytidine arabinoside [Ara-C], etoposide [VP-16], were also determined in the cytotoxic screens.

Figure 1 Structures of Nickel Complexes

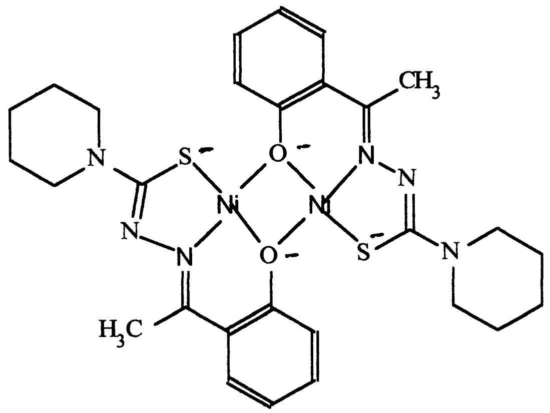

Compound **1** $[Ni(Appip)]_2$

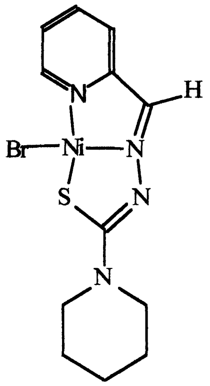

Compound **2** [Ni(Fopip)Br]

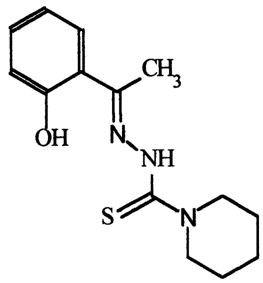

Compound **3** $H_2Appip$

Solid tumor cytotoxicity was determined by Liebovitz *et al.'s* method [9] utilizing 0.2% crystal violet/20% MeOH and read at 580 nm (Molecular Devices). *In vivo* anti-neoplastic activity in the Ehrlich ascites carcinoma was conducted in $CF_1$ male mice [30 g]. Compounds were administered at 8 mg/kg, I.P. on days 1-9. The animals were sacrificed on day 10, and the volume of tumor and astrocrit determined. 6-

Mercaptopurine was used as an internal standard. Percent inhibition of tumor growth was determined according to the literature [10].

**Incorporation Studies**

Compound **1** was selected for a mode of action study in L1210 leukemia cells based on its cytotoxic action. Incorporation of labeled precursors into $^3$H-DNA, $^3$H-RNA and $^3$H-protein for $10^6$ L1210 cells was obtained [11]. The concentration response at 10, 25, 50 and 100 μM required for inhibition of DNA, RNA and protein synthesis was determined for 60 min incubations. The incorporation of $^{14}$C-glycine (53.0 mCi/mmol) into purines was obtained by the method of Cadman *et al.* [12]. Incorporation of $^{14}$C-formate (53.0 mCi/mmol) into pyrimidines was determined by the method of Christopherson *et al* [13].

**Enzyme Assays**

Inhibition studies of various enzyme activities were performed by first preparing the appropriate L1210 cell homogenates or subcellular fractions, then adding the drug to be tested during the enzyme assay. For the concentration response studies, inhibition of enzyme activity was determined at 10, 25, 50 and 100 μM of compound **1**, after 60 min incubations. DNA polymerase α activity was determined in cytoplasmic extracts isolated by Eichler *et al.'s* method[14]. Nuclear DNA polymerase ß was determined by isolating nuclei [15]. The polymerase assay for both alpha and beta was described by Sawada *et al.*[16] with $^3$H-TTP. Messenger-, ribosomal- and transfer-RNA polymerase enzymes were isolated with different concentrations of ammonium sulfate; individual RNA polymerase activities were determined using $^3$H-UTP [17,18]. Ribonucleoside reductase activity was measured using $^{14}$C-CDP with dithioerythritol [19]. The deoxyribonucleotides $^{14}$C-dCDP were separated from the ribonucleotides by TLC on PEI plates. Thymidine, TMP and TDP kinase activities were determined using $^3$H-thymidine (58.3 mCi/mmol) in the medium of Maley and Ochoa [20]. Carbamyl phosphate synthetase activity was determined with the method of Kalman *et al.*[21]; citrulline was determined colorimetrically [22]. Aspartate transcarbamylase activity was measured by the method of Kalman *et al.*[21]; carbamyl aspartate was determined colorimetrically [23]. Thymidylate synthetase activity was analyzed by Kampf *et al.'s* method [24]. The $^3H_2O$ measured was proportional to the amount of TMP formed from $^3$H-dUMP. Dihydrofolate reductase activity was determined by the spectrophotometric method of Ho *et al.*[25]. PRPP amidotransferase activity was determined by Spassova *et al.'s* method [26]; IMP dehydrogenase activity was analyzed with 8-$^{14}$C-IMP (54 mCi/mmol) (Amersham, Arlington Heights, IL) after separating XMP on PEI plates (Fisher Scientific) by TLC [27]. Protein content was determined for the enzymatic assays by the Lowry technique[28]. After deoxyribonucleoside triphosphates were extracted [29], d[NTP] pool levels were determined by the method of Hunting and Henderson[30] with calf thymus DNA, E. coli DNA polymerase I, non-limiting amounts of the three deoxyribonucleoside triphosphates not being assayed, and either 0.4 mCi of ($^3$H-methyl)-dTTP or (5-$^3$H)-dCTP.

**DNA Studies**

The effects of compound **1** on DNA strand scission was determined by the methods of Suzuki *et al.*[31], Pera *et al.*[32] and Woynarowski *et al.*[32]. L1210 lymphoid leukemia cells were incubated with 10 μCi thymidine methyl-$^3$H, 84.0 Ci/mmol for 24 hr at 37°C. L1210 cells ($10^7$) were harvested and then centrifuged at 600 g X 10 min in PBS. They were later washed and suspended in 1 ml of PBS. Lysis buffer (0.5 ml; 0.5 M NaOH, 0.02 M EDTA, 0.01% Triton X-100 and 2.5% sucrose) was layered onto a 5-20% alkaline-sucrose gradient (5 ml; 0.3 M NaOH, 0.7 KCl and 0.01 M EDTA); this was followed by 0.2 ml of the cell preparation. After the gradient was incubated for 2.5 hr at room temperature, it was centrifuged at 12,000 RPM at 20°C for 60 min (Beckman rotor SW60). Fractions (0.2 ml) were collected from the bottom of the gradient, neutralized with 0.2 ml of 0.3 N HCl, and measured for radioactivity. Thermal calf thymus ct-DNA denaturation studies and ct-DNA viscosity studies were conducted after incubation of compound **1** at 100 μM at 37°C for 24 hr [33]. L1210 DNA topoisomerase II was isolated by the method of Liu et al. [34] and the activity was determined by the method of Rowe et al. [ 35].

**Statistics**

The mean and standard deviation are designated by "X ± SD." The probable level of significance (p) between test and control samples was determined by the Student's "t" test with the raw data.

### Results

The nickel complexes demonstrated mixed results in the Ehrlich ascites carcinoma screen compound **1** afforded 99.5% inhibition at 1 mg/kg/day, I.P. Compounds **2** and **3** caused 28% and 67% reduction of tumor growth at 8 mg/kg/day, I.P., respectively. Cytotoxicity was demonstrated by all three compounds against the growth of murine or human leukemias, human HeLa uterine suspended carcinoma, colon adenocarcinoma SW480, KB nasopharynx, lung MB 9812 bronchogenic carcinomas, but only compounds **1** and **2** were active against solid HeLa uterine carcinoma and rat osteosarcoma growth. The ED50 needs to be < 4 μg/ml for significant activity. The compounds were not active against the growth of human lung A 549, ileum adenocarcinoma HCT-8, and skin epidermoid A431 cell growth [data not shown].

Table 1 The Cytotoxicity of the Nickel Complexes [ ED50 = μg/ml ]

| | L1210 Lymphoid Leukemia | $Tmolt_3$ Leukemia T Cell | HeLa $S^3$ Uterine Carcinoma | HeLa Solid Uterine | Colon Adenocarci noma SW480 | KB Naso-pharynx | UMR Osteo-sarcoma | Lung MB-9812 |
|---|---|---|---|---|---|---|---|---|
| Cpd #1 | 1.32 | 3.59 | 2.97 | 1.82 | 2.14 | 3.62 | 3.67 | 4.02 |
| Cpd #2 | 1.81 | 1.02 | 2.76 | 3.50 | 1.13 | 1.82 | 2.46 | 0.77 |
| Cpd #3 | 2.98 | 3.56 | 1.37 | 7.34 | 2.72 | 2.78 | 6.35 | 2.21 |
| VP-16 | 1.83 | | 7.87 | 3.05 | 3.34 | 3.32 | 3.57 | 3.50 |
| 6MP | 2.43 | 1.62 | 2.12 | 5.61 | 3.61 | 11.04 | 9.13 | 4.29 |
| Ara-C | 2.42 | 2.67 | 2.13 | 4.74 | 342 | 2.84 | 0.86 | 6.16 |
| 5-FU | 1.41 | 1.41 | 2.47 | 4.11 | 3.09 | 1.25 | 3.52 | 5.64 |

The mode of action study with compound **1** in L1210 cells showed that DNA synthesis was reduced greater than 65% at 25 μM and 99% at 100 μM whereas RNA and protein syntheses were inhibited 37% and 34% respectively after 60 min at 100 μM of compound **1**. DNA polymerase α activity was reduced 55% and m-RNA polymerase activity was reduced 33% at 100 μM but r- and t-RNA polymerase activities were not affected by compound **1**. Ribonucleoside reductase activity was only reduced 20% but dihydrofolate reductase activity was inhibited 54% after 60 min at 100 μM. *De novo* purine synthesis was inhibited in a concentration dependent manner with 63% reduction at 100 μM. The activities of both regulatory enzymes of the pathway were reduced ; PRPP amido transferase activity by 56% and IMP dehydrogenase activity 54% by compound **1** . *De novo* pyrimidine synthesis was not significantly affected by compound **1** nor were the early regulatory enzymes of the pathway affected. Thymidylate synthetase activity was reduced 44% and thymidine-monophosphate kinase activity was reduced 49% at 100 μM. TDP kinase activity was elevated 185% by compound **1**. The d[GTP] pool level was reduced 13% and d[CTP] and d[TTP] pool levels were elevated 68-78% after 60 min incubation with compound **1**. L1210 DNA strand scission did occur with compound **1** after incubation for 24 hr. at 100 μM [Fig 2]. ct-DNA studies demonstrated that DNA viscosity was reduced from 323 sec for the control to 283 sec. for compound **1** indicating smaller fragments of DNA. The DNA thermal denaturation Tm value for the control was 87.5°C and was 92°C for compound **1**. L1210 DNA topoisomerase activity was not affected by compound **1** at 100 μM.

### Discussion

The nickel(II) complexes of thiosemicarbazones proved to be potent anti-neoplastic and cytotoxic agents. The complexes did demonstrate some specificity for select tumor growth inhibition. The two nickel compounds tested previously demonstrated significant activity in the ileum adenocarcinoma, and skin A431 screens and were more potent in the KB nasopharynx and osteosarcoma screens[1], but the two new nickel complexes **1** and **2** were more potent in the L1210 screen. The mode of action in L1210 leukemia

| Table 2 The Effects of the Nickel Complex on L1210 Nucleic Acid Metabolism | | | | |
|---|---|---|---|---|
| | | Percent of Control | | |
| N=6 | Control | | Compound 1 | |
| | | 25 uM | 50 uM | 100 uM |
| DNA synthesis | 100±5 | 33+4* | 28+4* | 1+1* |
| RNA synthesis | 100±6 | 133±7 | 77±5 | 63±5 |
| Protein synthesis | 100±4 | 116±9 | 83±6 | 66±4 |
| DNA polymerase a | 100±5 | 63±5 | 53±4 | 45±4 |
| m-RNA polymerase | 100±6 | 69±5 | 69±4 | 67+4 |
| r-RNA polymerase | 100±5 | 126±7 | 111±8 | 101±6 |
| t-RNA polymerase | 100±8 | 119±6 | 114±5 | 99±4 |
| Ribonucleoside reductase | 100±6 | 91+6 | 86+5 | 80+5 |
| Purine de novo synthesis | 100±7 | 95+5 | 50+5 | 37+4 |
| PPRP amido-transferase | 100±8 | 153+7 | 89+5 | 44+4 |
| IMP dehydrogenase | 100±5 | 70+6 | 68+5 | 46+3 |
| Pyrimidine de novo synthesis | 100±7 | 99+6 | 97+5 | 101+5 |
| Cambamyl phosphate synthetase | 100±7 | 101+6 | 115+6 | 127+7 |
| Aspartate transcarbamylase | 100±6 | 96+5 | 93+5 | 90+5 |
| Thymidine synthetase | 100±6 | 63+5 | 61+4 | 56+4 |
| Thymidine kinase | 100±6 | 158+8 | 137+6 | 85+5 |
| TMP kinase | 100±5 | 124+6 | 94+5 | 51+4 |
| TDP kinase | 100±5 | 142+7 | 250+6 | 285+6 |
| Dihydrofolate reductase | 100±4 | 82+6 | 68+5 | 46+4 |
| d(ATP) | 100±6 | | | 87+6 |
| d(GTP) | 100±6 | | | 100+5 |
| d(CTP) | 100±7 | | | 168+6 |
| d(TTP) | 100±5 | | | 178+7 |
| | | | | |

cell for the nickel complex was similar to that of the copper(II) complexes of thiosemicarbazones [2]. DNA synthesis was the major target of the nickel complexes with marginal effects on RNA and protein synthesis after 60 min. Inhibition of DNA polymerase α activity by the compound was one of the reasons DNA synthesis was suppressed. This effect on this enzyme was similar to that observation with the copper(II) complexes as were the mixed effects on the RNA polymerase activities. The copper(II) complexes did have a higher magnitude of inhibition for ribonucleoside reductase activity[1,2] than the nickel compound; however, inhibition of this enzyme would be additive with the overall reduction of DNA synthesis since there would be a reduction in the deoxyribonucleosides and deoxyribonucleotides in the nucleus for their incorporation into DNA.

A major site of inhibition of the nickel complex was in the purine pathway of L1210 cells. Both regulatory enzymes were markedly reduced in activity, similar to the effects afforded by 6-MP. This would lead to reduced deoxyribopurines as well as inhibition of DNA synthesis and cell death. There were not apparent blocks by the agent in the pyrimidine pathway, although some inhibition of thymidylate synthetase activity occurred. The elevation of TDP-kinase activity by the nickel complex may help explain the observed elevation of deoxyribopyrimidine pools. On the other hand, the d[NTP] pools would be elevated in the cell if DNA polymerase α was inhibited in that the deoxytriphosphate nucleotides were not incorporated into the new strand of DNA. The nickel complex did cause fragmentation of DNA but did not cause alkylation of bases of DNA or intercalation between base pairs. The nickel complex did not inhibit L1210 DNA topoisomerase II activity. The copper complexes of thiosemicarbazones were potent inhibitors of L1210 DNA topoisomerase II activity and were able to cause DNA protein linked breaks[2].

Figure 2 The L1210 DNA Strand Scission

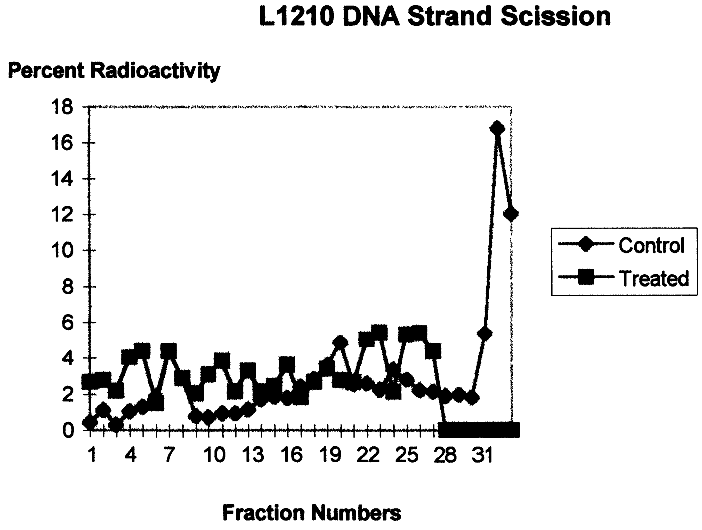

### References

1. Hall IH, Rajendran KG, West DX, Liberta AE. Anti-Cancer Drugs 1993; 4:231.
2. West DX, Liberta AE, Rajendran KG, Hall IH. Anti-Cancer Drugs 1993; 4: 241
3. Miller MC III, Bastow KF, Stineman CN, Vance JR, Song SS, West DX, Hall IH, Appl. Org Met. Chem. In preparation.
4. Miller MC III, Stineman CN, Vance JR, West DX, Hall IH. Anti-Cancer Res. In preparation.
5. Miller MC III, Stineman CN, Vance JR, West DX, Hall IH. In preparation.
6. West DX, Yang Y, Klein TL, Goldberg KI, Liberta AE. Polyhedron1995; 14, 3051.
7. West DX, Ooms CE, Saleda JS, Gebrmedhin H, Liberta AE. Transition Metal Chem 1994; 19, 554.
7. Huang Y, Hall IH, Anticancer Res. 1996; 16: 597.
8.Geran RJ, Greenburg NH, MacDonald MM, Schumacher AM, Abbott BJ. Cancer Chemo Rep 1972; 3: 9.
9. Leibovitz AL, Stinson JC, McComb WB III, McCoy CE, Mazur KC, Mabry ND. Cancer Res 1976; 36: 4562.
10. Piantadosi C, Kim CS, Irving JL. J. Pharm. Sci. 1969; 58: 821.
11. Liao LL, Kupchan SM, Horwitz SB. Mol Pharmacol 1976; 12: 167.
12. Cadman E, Heimer R, Benz C. J Biol Chem 1981; 256: 1695.
13. Christopherson RI, Yu ML, Jones ME. Anal Biochem 1981; 11: 240.
14. Eichler DC, Fisher PA, Korn D. J Biol Chem 1977; 252: 4011.
15. Sawada H, Tatsumi K, Sadada M, Shirakawa S, Nakamura T, Wakisaka G. Cancer Res 1974; 34: 3341.
16. Anderson KM, Mendelson IS, Guzik G. Biochem Biophys Acta 1975; 383: 56.
17. Hall IH, Carlson GL, Abernathy GS, Piantadosi C. J Med Chem 1974; 17: 1253-1257.
18. Moore EC, Hurlbert RB. J Biol Chem 1966; 241: 4802.
19. Maley F, Ochoa S. J Biol Chem 1958; 233: 1538.
20. Spassova MK, Russev GC, Goovinsky EV. Biochem Pharmacol 1976; 25: 923.
21. Becker JH, Lohr GW. Klin Wochenschr 1979; 57: 1109.
22. Kalman SM, Duffield PH, Brzozwki TJ. J Biol Chem 1966; 241: 871.
23. Archibald RM. J Biol Chem 1944; 156: 121.
24. Koritz SB, Gohen PP. J Biol Chem 1954; 209: 145.
25. Kampf A, Barfknecht RL, Schaffer PJ, Osaki S, Mertes MP. J Med Chem 1976; 19: 903.
26. Ho YK, Hakala T, Zakrzewski SF. Cancer Res 1971; 32: 1023.
27. Lowry OH, Rosebrough J, Farr AL, Randall RJ. J Biol Chem 1951; 193: 265.
28. Bagnara AS, Finch LR. Anal Biochem 1971; 45: 24.

29 Hunting D, Henderson JF. Can J Biochem 1982; 59: 723.
30. Suzuki H, Nishimura T, Muto SK Tanaka N. J Antibacteriol 1978; 32: 875.
31. Pera JF Sr, Rawlings CJ, Shackleton J, Roberts JJ. Biochem Biophy Acta 1981; 655: 152.
32. Woynarowski JW, Beerman TA, Konopa J. Biochem Pharmacol 1981; 30: 3005.
33. Zhao Y, Hall IH, Oswald CB, Yokoi T, Lee KH. Chem Phar Bull 1987; 35: 2052.
34. Liu LF, Rowe TC, Yang L, Tewey KM, Chen GL. J. Biol. Chem. 258:15365-15370, 1963.
35. Rowe TC, KM Tewey, Liu LF. J. Biol. Chem. 259: 9177-9181, 1964.

**Received: February 27, 1997 - Accepted: March 10, 1997 - Received in revised camera-ready format: March 26, 1997**

